# Management of Anesthesia under Extracorporeal Cardiopulmonary Support in an Infant with Severe Subglottic Stenosis

**DOI:** 10.1155/2016/6871565

**Published:** 2016-02-18

**Authors:** Rie Soeda, Fumika Taniguchi, Maiko Sawada, Saeko Hamaoka, Masayuki Shibasaki, Yasufumi Nakajima, Satoru Hashimoto, Teiji Sawa, Yoshinobu Nakayama

**Affiliations:** ^1^Department of Anesthesiology, Kyoto Prefectural University of Medicine, 465 Kajii-cho, Kamigyo, Kyoto 602-8566, Japan; ^2^Department of Anesthesiology, Kansai Medical University, 2-5-1 Shin-machi, Hirakata, Osaka 573-1010, Japan; ^3^Division of Critical Care, Kyoto Prefectural University of Medicine Hospital, 465 Kajii-cho, Kamigyo, Kyoto 602-8566, Japan

## Abstract

A 4-month-old female infant who weighed 3.57 kg with severe subglottic stenosis underwent tracheostomy under extracorporeal cardiopulmonary support. First, we set up extracorporeal cardiopulmonary support to the infant and then successfully intubated an endotracheal tube with a 2.5 mm inner diameter before tracheostomy by otolaryngologists. Extracorporeal cardiopulmonary support is an alternative for maintenance of oxygenation in difficult airway management in infants.

## 1. Introduction

We experienced difficult airway management under extracorporeal cardiopulmonary support in an infant with severe subglottic stenosis. An algorithm of difficult airway management for infants with subglottic stenosis should effectively include extracorporeal cardiopulmonary support. This support includes cardiopulmonary bypass (CPB) and/or extracorporeal membrane oxygenation (ECMO) to maintain blood gas exchange during insecure tracheal intubation and difficult tracheostomy in infants with subglottic airway stenosis.

## 2. Case Presentation

**Figure 1:**
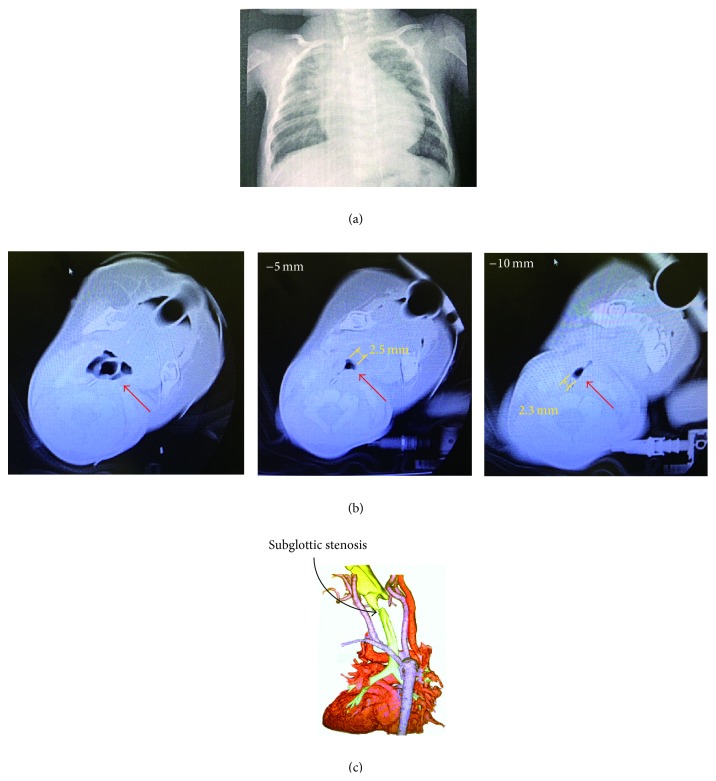
Chest X-ray posterior-anterior view and computed tomographic images. (a) Chest X-ray posterior-anterior (PA) view. (b) Computed tomographic (CT) images of the neck region (every 5 mm). Left panel: level of the larynx. Center panel: −5 mm from the level of the larynx. Right panel: −10 mm from the level of the larynx. (c) Three-dimensional CT reconstruction image of the larynx and vessels. The arrow shows subglottic stenosis.

A 4-month-old female infant, who weighed 3.57 kg and was 54.6 cm in height, was hospitalized because of the diagnosis of severe subglottic stenosis. At birth, she was diagnosed with 22q11.2 deletion syndrome with multiple anomalies, including thymic aplasia, aortic arch interruption, ventricular septal defect, atrial septal defect, and subvalvular aortic stenosis. Eight days after birth, when she weighed 2.77 kg and was 46 cm in height, she had surgery of the right pulmonary artery banding under general anesthesia. This anesthesia management allowed easy intubation (Cormack-Lehane Grade I) with a cuffed endotracheal tube (3.0 mm inner diameter (ID)). She was extubated at the pediatric intensive care unit (PICU) without any complications the next day after surgery. Three weeks later, she had secondary radical surgery of the aortic arch and intracardiac repair under general anesthesia with intubation with a cuffed endotracheal tube (3.0 mm ID) without any problems. After the second surgery, she was under artificial ventilation in the PICU. On the 5th postsurgical day, she was extubated and placed under bubble continuous positive airway pressure (bCPAP). However, after extubation, she had stridor in her respiration and had difficulty weaning from bCPAP for the next 3 days. At the 7th postsurgical day, an otolaryngologist performed nasolaryngeal optical fiberscopy, and she was diagnosed with right vocal cord paralysis. Six weeks after surgery, she was discharged from the hospital and was placed at home care. However, 6 weeks after discharge, at 4 months old, she was rehospitalized because of respiratory distress with the constriction situation at the time of crying and reduced suckling force at breastfeeding. Blood arterial gas analysis showed hyper apnea and metabolic alkalosis as follows: pH, 7.45; PaO_2_, 67.8 mmHg; PaCO_2_, 54.9 mmHg; base excess, 11.7; lactate level, 1.0 mEq/L; and SaO_2_, 95.4% at room air. Severe subglottic stenosis with an estimated diameter of 2.3–2.5 mm in the narrowest portion of her larynx was diagnosed by computed tomography (Figure 1). Emergent tracheostomy was then planned under general anesthesia, and the method of induction of anesthesia was discussed.

**Figure 2:**
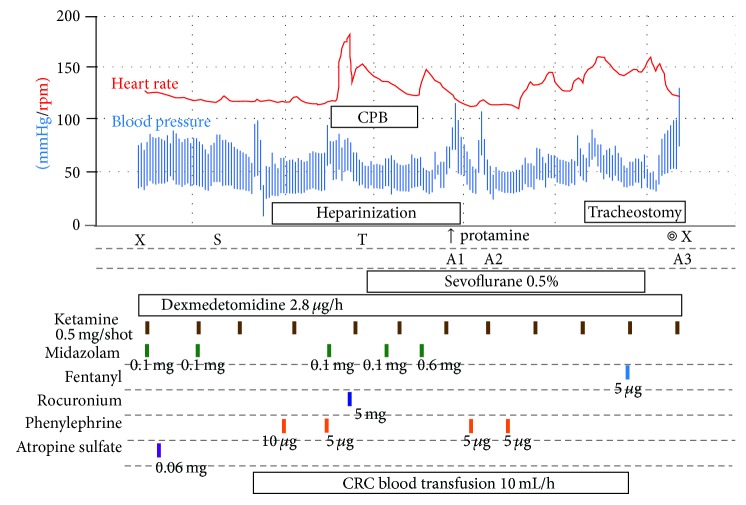
Time course of anesthesia management under extracorporeal cardiopulmonary support and the following tracheostomy. X: start and end of anesthesia management. S: start of surgery for extracorporeal cardiopulmonary support. T: tracheal intubation. Arterial blood gas data were measured at A1, A2, and A3. A1: pH, 7.46; PaCO_2_, 36.8 mmHg; PaO_2_, 240 mmHg; base excess, 2.4 mmol/L; and SaO_2_, 100%. A2: pH, 7.39; PaCO_2_, 46.8 mmHg; PaO_2_, 118 mmHg; base excess, 2.8 mmol/L; and SaO_2_, 99%. A3: pH, 7.49; PaCO_2_, 33.3 mmHg; PaO_2_, 509 mmHg; base excess, 2.9 mmol/L; and SaO_2_, 100%.

Because the patient just maintained spontaneous ventilation with depression of epigastric intercostal, there was a high risk of “cannot intubate cannot ventilate” during induction of anesthesia under the method of being either awake or anesthetized during induction. Therefore, in advance of the procedure of tracheal intubation, we decided to keep her under spontaneous ventilation and performed extracorporeal cardiopulmonary support under regional anesthesia with light sedation (Figure 2). Venoarterial extracorporeal cardiopulmonary support with cannulation by the open cut method was performed by cardiac surgeons in case of resuscitation with cardiac support. She was sedated with dexmedetomidine (0.7 *µ*g/kg/h), ketamine 1.0 mg (0.5 mg, 2 shots), and midazolam 0.2 mg (0.1 mg, twice). Total heparinization followed by arterial and venous cannulation of the extracorporeal circuit to the right femoral artery and right femoral vein was then performed under local anesthesia with 1% lidocaine (1.7 mL). Additional administration of ketamine (0.5 mg, 2 shots) and midazolam (0.1 mg, once) was performed after the extracorporeal circuit was started and oxygenation of arterial blood was attained by evaluation using pulse oximetry. Semiawake tracheal intubation was carried out with an uncuffed endotracheal tube (2.5 mm ID). Although slight resistance was felt at the time the tip of the endotracheal tube passed through the narrowest region of the subglottis, the tube was successfully placed in an appropriate position with a depth of 8.5 cm from the infant's mouth. A total of 5 mg rocuronium bromide was administered intravenously and respiration of the patient was under the control of positive airway pressure ventilation. Ten minutes after tracheal intubation, extracorporeal cardiopulmonary support was stopped and decannulation and neutralization of heparin with protamine were carried out. Tracheostomy was successfully performed by an otolaryngologist in the next 20 min. The anesthesia time was 3 hours and 8 minutes and the extracorporeal cardiopulmonary support time was 19 minutes. The patient was admitted to the PICU for postoperative management.

## 3. Discussion

For more than 50 years, anesthesia management of an infant who has subglottic stenosis has been discussed [1–3]. Although subglottic stenosis can occur in all age groups, pediatric cases can be pathophysiologically divided into two categories, which are congenital and acquired. Our patient was diagnosed with 22q11.2 deletion syndrome (22q11.2DS), which is also known as DiGeorge syndrome, DiGeorge anomaly, and velocardiofacial syndrome. This syndrome is caused by deletion of a small piece of chromosome 22 with five typical symptoms, such as cardiac defects, abnormal facies, thymic hypoplasia, cleft palate, and hypocalcemia [4, 5]. In our case, radical repair of aortic arch interruption probably caused right vocal cord paralysis due to recurrent nerve injury, which was eventually associated with the occurrence of acquired subglottic stenosis.

Various research groups have proposed guidelines or an algorithm for difficult airway management in pediatrics [6–9]. Among them, the pediatric difficult airway guidelines by the Guidelines Group, which is supported by the Association of Paediatric Anaesthetists, the Difficult Airway Society, are the most popular [7–9]. Three guidelines relate to the management of a difficult airway in children aged 1 to 8 years as follows: APA1 is difficult mask ventilation during routine induction of anesthesia; APA2 is unanticipated difficult tracheal intubation during routine induction of anesthesia; and APA3 is cannot intubate cannot ventilate in paralyzed, anesthetized patients. However, these guidelines are for patients aged older than 1 year, and difficult airway cases in pediatrics frequently occur under 1 year of age. Recently, we experienced three different infant cases with difficult airway management, including this case. One case was a newborn who had large tumors extruding out of his mouth and mask ventilation was difficult to perform in induction of anesthesia [10]. Another case was a girl with a malignant rhabdoid tumor in her oral cavity and she was difficult to intubate through oral access [11]. Therefore, guidance or an algorithm for management of a difficult airway in children aged younger than 1 year is required separately from the currently available guidelines for patients who are aged older than 1 year.

**Figure 3:**
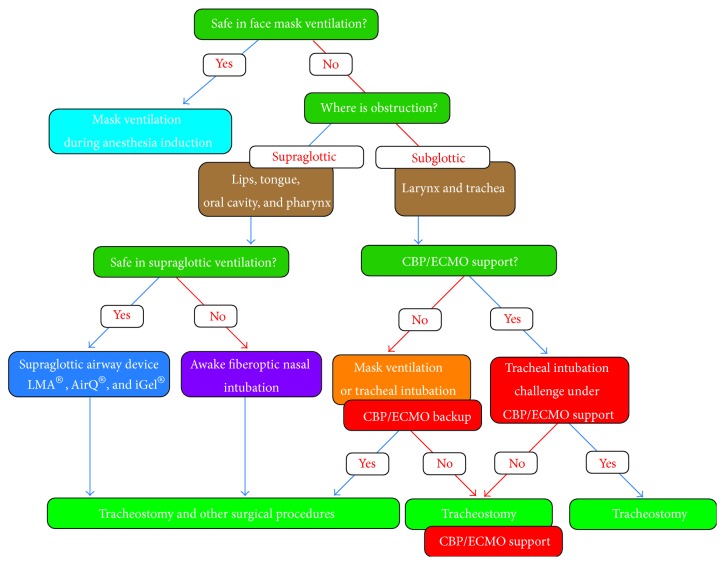
Algorithm for anticipated difficult airway management for infants. Supraglottic airway devices and awake nasal fiberoptic intubation, as well as extracorporeal cardiopulmonary support or extracorporeal cardiopulmonary backup support, are incorporated into the algorithm.

During induction of anesthesia for tracheostomy in a pediatric subglottic stenosis patient, gas exchange can be maintained in one of five ways [12]: (1) jet ventilation, (2) distal tracheal intubation and intermittent positive pressure ventilation, (3) spontaneous ventilation, (4) mask ventilation (and tracheal intubation) under backup support of extracorporeal cardiopulmonary, including CPB and ECMO, and (5) intubation challenge (and tracheostomy) under CPB or ECMO support. CPB and ECMO, both of which can be a life-saving maneuver for near or total occlusion of the airway, have been used for tracheal surgery [13–16]. Tracheostomy in infants with subglottic stenosis is a challenging approach, even for experienced otolaryngologists [17, 18]. In addition, because there is a potential risk of excessive bleeding due to anticoagulation requirements, tracheostomy should be performed only after establishment of an airway on induction under the support (or the backup support) of these devices. In our case, tracheal intubation was safely carried out under the support of CPB. After CPB was terminated and anticoagulation was neutralized, tracheostomy was performed under safe conditions to avoid the risk of bleeding at the surgical site. Instead of tracheal intubation challenge under the direct support of CPB, mask ventilation or tracheal intubation challenge under backup support of CPB for emergent oxygenation was an alternative choice in the airway management of this case. However, in this case, we chose a safer oxygenation strategy because the good support of an experienced pediatric cardiac surgery team was available in our facility. We think that, regardless of direct support or backup support, CPB and/or ECMO should be incorporated into the algorithm of anticipated difficult airway management for infants as an alternative procedure (Figure 3).

In conclusion, under extracorporeal cardiopulmonary support, we successfully managed anesthesia for tracheostomy in an infant with severe subglottic stenosis. CPB and/or ECMO should be incorporated into the algorithm of anticipated difficult airway management for infants.
